# BI-2 destabilizes HIV-1 cores during infection and Prevents Binding of CPSF6 to the HIV-1 Capsid

**DOI:** 10.1186/s12977-014-0120-x

**Published:** 2014-12-11

**Authors:** Thomas Fricke, Cindy Buffone, Silvana Opp, Jose Valle-Casuso, Felipe Diaz-Griffero

**Affiliations:** Department of Microbiology and Immunology, Albert Einstein College of Medicine Bronx, Bronx, NY 10461 USA; Albert Einstein College of Medicine, 1301 Morris Park – Price Center 501, New York, NY 10461 USA

**Keywords:** HIV-1, BI-2, PF74, Capsid, Stability, Uncoating, CPSF6

## Abstract

**Background:**

The recently discovered small-molecule BI-2 potently blocks HIV-1 infection. BI-2 binds to the N-terminal domain of HIV-1 capsid. BI-2 utilizes the same capsid pocket used by the small molecule PF74. Although both drugs bind to the same pocket, it has been proposed that BI-2 uses a different mechanism to block HIV-1 infection when compared to PF74.

**Findings:**

This work demonstrates that BI-2 destabilizes the HIV-1 core during infection, and prevents the binding of the cellular factor CPSF6 to the HIV-1 core.

**Conclusions:**

Overall this short-form paper suggests that BI-2 is using a similar mechanism to the one used by PF74 to block HIV-1 infection.

## Findings

The ability of the novel HIV-1 inhibitor BI-2 to potently block HIV-1 infection has been correlated with stabilization of in vitro assembled HIV-1 CA-NC complexes [1-3]. Crystal structure of the drug with the N-terminal domain of capsid (CA_NTD_) revealed that BI-2 binds in the site 2 pocket [1], as it has been shown for the small-molecule inhibitor PF74 [1,4,5]. Using a novel capsid stability assay, we have demonstrated that BI-2 and PF74 stabilize in vitro assembled HIV-1 capsid-nucleocapsid (CA-NC) complexes [2]. Counter intuitively, PF74 destabilizes the HIV-1 core during infection of cells [5]. In addition, several reports have demonstrated that PF74 prevents the binding of the cellular factor cleavage and polyadenylation specific factor 6 (CPSF6) to the viral capsid [2,6]. Previous observations have shown that BI-2 stabilizes in vitro assembled HIV-1 CA-NC complexes by using two different assays [1,2]. Because BI-2 has been suggested to inhibit HIV-1 infection, at least in part, by stabilizing the viral capsid [1,2], we investigated the effects of BI-2 in infection by analyzing 1) HIV-1 DNA metabolism, 2) the fate of the HIV-1 capsid, 3) binding of CPSF6 to HIV-1 capsid, and 4) the ability of BI-2 to block infection by other retroviruses.

### BI-2 blocks infection of HIV-1 after reverse transcription but prior to nuclear import

**Figure 1:**
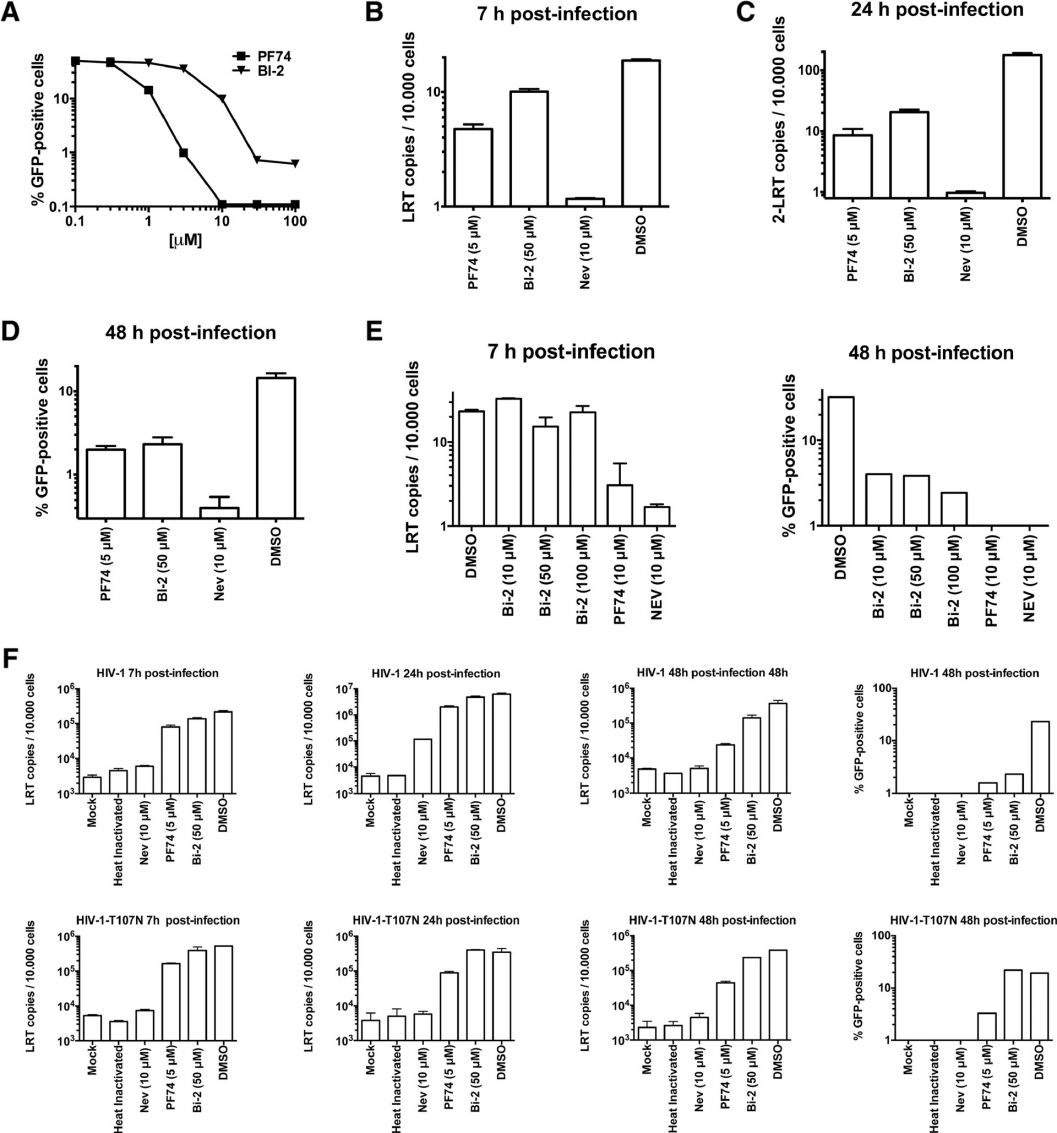
**BI-2 blocks the formation of 2-LTR circles during HIV-1 infection.** Cf2Th cells were challenged with HIV-1 expressing GFP as a reporter (HIV-1-GFP) in the presence of increasing concentrations of BI-2 or PF74. Infection was determined 48 hours post-infection by measuring the percentage of GFP-positive cells by flow cytometry **(A)**. Similar results were obtained in three independent experiments and a representative experiment is shown. Similarly, Cf2Th cells treated with BI-2, PF74 or DMSO were challenged with DNAse-pretreated HIV-1-GFP viruses. Subsequently, cells were harvested 7, 24 and 48 hours post-infection to measure HIV-1 late reverse transcripts (LRT) **(B)**, formation of HIV-1 2-LTR circles **(C)** and infectivity **(D)**, respectively. As control, we performed similar measurements in the presence of the reverse transcriptase inhibitor nevirapine (Nev) **(B-D)**. Similar results were obtained in three independent experiments and standard deviations are shown. **(E)** Similarly, Cf2Th cells treated with the indicated concentrations of BI-2 were challenged with DNAse-pretreated HIV-1-GFP viruses. Subsequently, cells were harvested 7 and 48 hours post-infection to measure HIV-1 LRT (left panel) and infection (right panel), respectively. As a control, we performed similar experiments in the presence of the reverse transcriptase inhibitor nevirapine (Nev). Similar results were obtained in three independent experiments and standard deviations are shown. **(F)** Formation of Late reverse transcripts by HIV-1-GFP and HIV-1-T107N-GFP were measured at 7, 24 and 48 hours post-infection in the presence of the indicated amounts of BI-2 or PF-74. Viral Infection was determined by counting the percentage of GFP-positive cells 48 hours post-infection. Similar results were obtained in three independent experiments and standard deviations are shown.

We initially studied the ability of BI-2 to block HIV-1-GFP infection in canine Cf2Th cells at the indicated concentrations (Figure 1A). As a control, we performed similar experiments using the small-molecule PF74 [1,2,4,5]. Our experiments showed that 50 μM of BI-2 is equivalent to 5 μM of PF74 when comparing inhibition of HIV-1-GFP infection (Figure 1A). These drugs did not exhibit cellular toxicity at the used concentrations, as determined by propidium iodide exclusion [7]. Next we challenged dog Cf2Th cells with similar amounts of HIV-1-GFP in the presence of BI-2. Infections were harvested at 7, 24 and 48 hours post-infection to analyze late reverse transcripts (LRT) (B), formation of 2-LTR circles (C) and infectivity (D), respectively. As a control, we performed similar infections in the presence of DMSO. To control for a block in reverse transcription, we used the inhibitor nevirapine [8], which completely blocks HIV-1-GFP reverse transcription (Figure 1B). BI-2 did not affect the occurrence of reverse transcription when compared to the effect of nevirapine (Figure 1B); this result is reminiscent of the effect of the related small molecule BI-1 to reverse transcription [1]. However, BI-2 potently blocked the formation of 2-LTR circles (Figure 1C). These results indicated that BI-2 blocks HIV-1-GFP infection after reverse transcription but prior to nuclear import, as demonstrated for BI-1 [1]. PF74 had a greater effect on the occurrence of reverse transcription when compared to BI-2, and potently blocked the formation of 2-LTR circles (Figure 1B-C), as previously shown [4,5]. Inhibition of HIV-1-GFP infection by BI-2 was comparable to PF74 at the indicated concentrations (Figure 1D). Previous observations showed that BI-1, a similar molecule to BI-2, did not affected the occurrence of reverse transcription [1]. Next we measured occurrence of HIV-1 reverse transcription in the presence of different concentrations of BI-2. To this end, we challenged dog Cf2Th cells with similar amounts of HIV-1-GFP in the presence of the indicated concentrations of BI-2, and measured the occurrence of reverse transcription and infection at 7 and 48 hours post-infection, respectively (Figure 1E). In agreement with previous findings using BI-1 [1], these experiments showed that BI-2 does not affect the occurrence of reverse transcription. As a control, we performed similar infections in the presence of nevirapine (Figure 1E), an inhibitor of reverse transcription. In addition, we monitored HIV-1 and HIV-1-T107N LRTs at 7, 24, and 48 hours post-infection in the presence of BI-2 or PF-74 (Figure 1F). Similarly, we found that BI-2 did not affect the formation of HIV-1 LRTs. In addition, BI-2 did not affect the formation of LRTs by HIV-1-T107N.

### BI-2 destabilizes the HIV-1 core during infection

**Figure 2:**
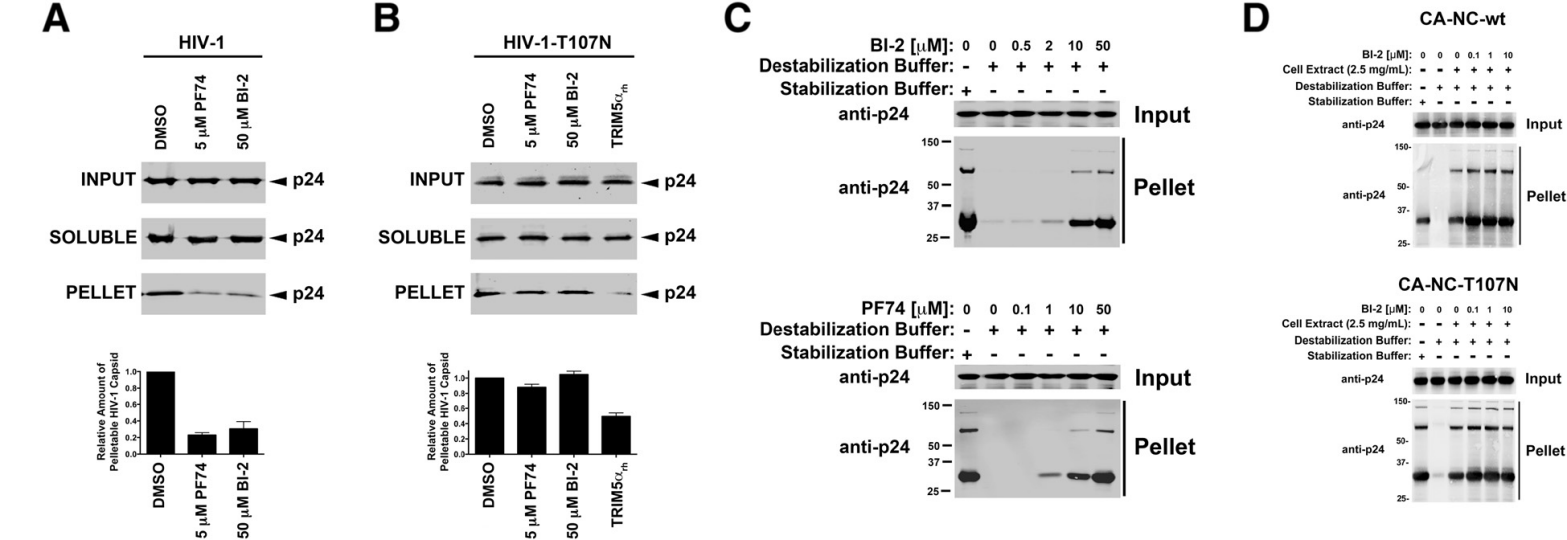
**BI-2 destabilizes the HIV-1 Core during infection. (A)** Cf2Th cells were challenged with HIV-1-GFP viruses in the presence of BI-2, and used to perform the fate of the capsid assay 12 hours post-infection, as described [9,11]. Briefly, HIV-1 infected cells were used to prepare post-nuclear supernatants that were layered onto a 50% sucrose cushion to separate soluble from pelletable HIV-1 capsids. INPUT, SOLUBLE and PELLET fractions were analyzed by Western blotting using antibodies against HIV-1 CA p24. As control, we studied the fate of the HIV-1 capsid in the presence of PF74. The percentages of pelletable capsids relative to the infected control in the presence of DMSO are shown. Similar results were obtained in three independent experiments and standard deviations are shown. **(B)** Cf2Th cells were challenged with HIV-1-GFP viruses bearing the capsid change T107N in the presence of BI-2, and used to perform the fate of the capsid assay 12 hours post-infection. As a control, we performed experiments in cells stably expressing rhesus TRIM5α.The percentages of pelletable capsids relative to the infected control in the presence of DMSO are shown. Similar results were obtained in three independent experiments and standard deviations are shown. **(C)** Stability of in vitro assembled HIV-1 CA-NC complexes in destabilization buffer containing increasing concentrations of BI-2 (upper panel) or PF74 (lower panel) were measured as described [2]. Input and Pellet fractions were analyzed by Western blotting using antibodies against HIV-1 CA p24. As control, stability of in vitro assembled HIV-1 CA-NC complexes in stabilization buffer was measured. Similar results were obtained in three independent experiments. **(D)** Stability of wild type (upper panel) or T107N mutant (lower panel) in vitro assembled HIV-1 CA-NC complexes in stabilization buffer containing cell extracts at increasing concentrations of BI-2 was measured, as described [2]. Similar results were obtained in three independent experiments.

We investigated the fate of the HIV-1 capsid in the presence of BI-2. For this purpose, we challenged Cf2Th cells with HIV-1-GFP in the presence of 50 μM BI-2 and performed the fate of the capsid 12 hours post-infection, as previously described [9-11]. As shown in Figure 2A, the use of BI-2 destabilized the HIV-1 core during infection when compared with the DMSO control. As a control, we used 5 μM PF74 that destabilized the HIV-1 core (Figure 2A), as previously shown [5]. Our results suggested that BI-2, like PF74, destabilizes the HIV-1 core during infection. To show that the destabilization of the HIV-1 core observed in the presence of BI-2 is specific to capsid, we performed the fate of the capsid assay using an HIV-1-GFP virus bearing the mutation T107N, which confers HIV-1 resistance to BI-2 and PF74 [1,4]. As shown in Figure 2B, BI-2 and PF74 did not affect the stability of the HIV-1 core bearing the change T107N. These results suggested that the ability of these drugs to destabilize the HIV-1 core is specific to capsid. As a control, we showed that TRIM5α_rh_ destabilizes the HIV-1 core (Figure 2B), as previously shown [12,13]. Next, we tested the ability of BI-2 to stabilize in vitro assembled HIV-1 CA-NC complexes using our previously published assay [2]. As we have previously shown, BI-2 as well as PF74 stabilize HIV-1 CA-NC complexes (Figure 2C) [2]. These results showed that BI-2, like PF74, stabilizes in vitro assembled HIV-1 CA-NC complexes, which is in agreement with previous reports [1,2]. Contrary to in vitro assembled HIV-1 CA-NC complexes that are mainly composed of capsid hexamers [14], the HIV-1 core is composed of capsid pentamers and hexamers [15,16]. The mature fullerene core is an assembly of capsid subunits displaying multiple quasi-equivalent conformations, which arise in part from the flexibility between the N-terminal and C-terminal domains of capsid. These multiple quasi-equivalent conformations result in the formation of hexamers and pentamers, which allow the formation of a curved capsid lattice. One possibility is that BI-2 and PF-74 limits the flexibility of the capsid to a range compatible only with the formation of hexamers; this might be the reason that BI-2 and PF74 stabilize in vitro assembled HIV-1 CA-NC complexes but destabilize the HIV-1 core during infection. A second possibility is that BI-2 requires the presence of cellular factors in order to destabilize in vitro assembled HIV-1 CA-NC complexes. To rule out that the ability of BI-2 to destabilize capsid complexes depends upon the presence of cellular factors, we tested the ability of BI-2 to destabilize in vitro assembled HIV-1 CA-NC complexes in the presence of cellular extracts. As shown in Figure 2D, the presence of cellular extracts did not alter the ability of BI-2 to destabilize capsid. As a control, similar experiments were performed using the capsid mutant T107N, which is resistant to BI-2 (Figure 2D). Future structural studies will shed light on this discrepancy.

### BI-2 prevents the binding of CPSF6 to in vitro assembled HIV-1 CA-NC complexes

**Figure 3:**
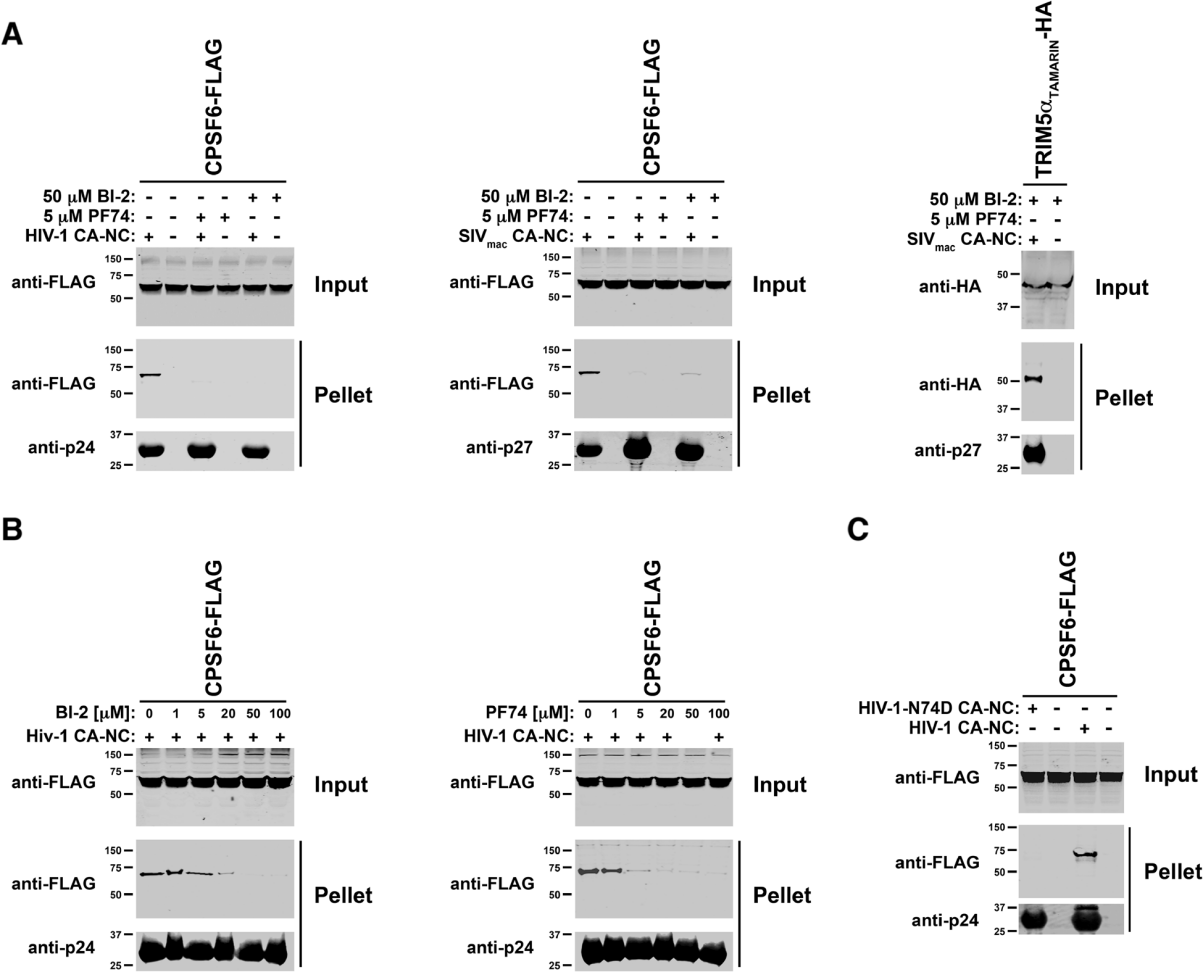
**BI-2 prevents the binding of the cellular factor CPSF6 to HIV-1 CA-NC complexes. (A)** The ability of CPSF6 to bind in vitro assembled HIV-1 CA-NC complexes in the presence of BI-2 was analyzed as previously described [22]. Briefly, extracts of 293 T cells transiently transfected with a CPSF6-FLAG construct (Input) were incubated with in vitro assembled HIV-1 or SIV_mac_ CA-NC complexes in the presence BI-2 for 1 h. Subsequently, extracts were applied onto 70% sucrose cushion and centrifuged, and the pelleted fraction was collected (Pellet). Input and Pellet fractions were analyzed using anti-FLAG and anti-p24 antibodies. As control, the binding of CPSF6 to in vitro assembled HIV-1 CA-NC complexes was studied in the presence of PF74. Similar results were obtained in three independent experiments and a representative experiments is shown. To control for the bona fide origin of in vitro assembled SIV_mac_ CA-NC complexes, we tested the ability of TRIM5α protein from Tamarin monkeys tagged with an HA epitope (TRIM5α_Tamarin_-HA) to bind SIV_mac_ capsid. **(B)** The ability of CPSF6 to bind in vitro assembled HIV-1 CA-NC complexes in the presence of increasing concentrations of BI-2 was analyzed (left panel). As a control, similar experiments were performed using increasing concentrations of PF74 (right panel). Similar results were obtained in three independent experiments and a representative experiments is shown. **(C)** To show that the interaction of CPSF6 with in vitro assembled HIV-1 CA-NC complexes is only dependent upon the capsid protein, we tested the ability of CPSF6 to bind in vitro assembled HIV-1 CA-NC complexes bearing the change N74D. Similar results were obtained in three independent experiments and a representative experiments is shown.

Expression of CPSF6 is required for the HIV-1 infection phenotype observed in human TNPO3-depleted cells [17-19]. We and others have previously demonstrated that the small-molecule HIV-1-inhibitor PF74 prevents the binding of CPSF6 to HIV-1 capsid [6,17]. Because of the similar phenotypes observed for HIV-1-GFP infection when using BI-2 and PF74, we tested the ability CPSF6 to bind in vitro assembled HIV-1 CA-NC complexes in the presence of BI-2. As shown in Figure 3A, BI-2 prevents the ability of CPSF6 to bind in vitro assembled HIV-1 CA-NC complexes. As previously shown, PF74 also prevented the binding of CPSF6 to in vitro assembled HIV-1 CA-NC complexes [17]. Interestingly, BI-2 and PF74 also inhibited the binding of CPSF6 to in vitro assembled simian immunodeficiency virus (SIV_mac_) CA-NC complexes (Figure 3A). As a control to show the bona fide origin of the SIV_mac_ capsid, we showed that TRIM5α from tamarin monkeys binds to in vitro assembled SIV_mac_ CA-NC complexes (Figure 3A) [20]. These results suggested that BI-2 prevents the binding of CPSF6 to the HIV-1 and SIV_mac_ cores. Next, we performed a dose response curve to better understand the ability of BI-2 to prevent the binding of CPSF6 to in vitro assembled HIV-1 CA-NC complexes. As shown in Figure 3B, we observed that using BI-2 at 50 μM completely inhibit the binding of CPSF6 to in vitro assembled HIV-1 CA-NC complexes. For comparison, we showed a dose response curve to understand the ability of PF74 to interfere with the binding of CPSF6 to in vitro assembled HIV-1 CA-NC complexes. As we have previously shown using PF74 at 5 μM inhibited the ability of CPSF6 to bind in vitro assembled HIV-1 CA-NC complexes [17]. Altogether these results showed that BI-2 prevents the ability of CPSF6 to interact with the HIV-1 core. Because CPSF6 binds to nucleic acids, and HIV-1 CA-NC complexes are assembled in the presence of nucleic acids [21], we performed a control to demonstrate that the interaction of CPSF6 with in vitro assembled HIV-1 CA-NC complexes is only dependent upon the capsid protein. To this end, we tested the ability of CPSF6 to bind in vitro assembled HIV-1 CA-NC complexes bearing the change N74D, which confers HIV-1 resistance to the overexpression of cytosolic CPSF6 [17,18]. As shown in Figure 3C, CPSF6 did not bind to vitro assembled HIV-1 CA-NC complexes bearing the change N74D. These results indicated that CPSF6 is specifically binding to capsid, as shown [17].

### Ability of BI-2 to block infection by different retroviruses

**Figure 4:**
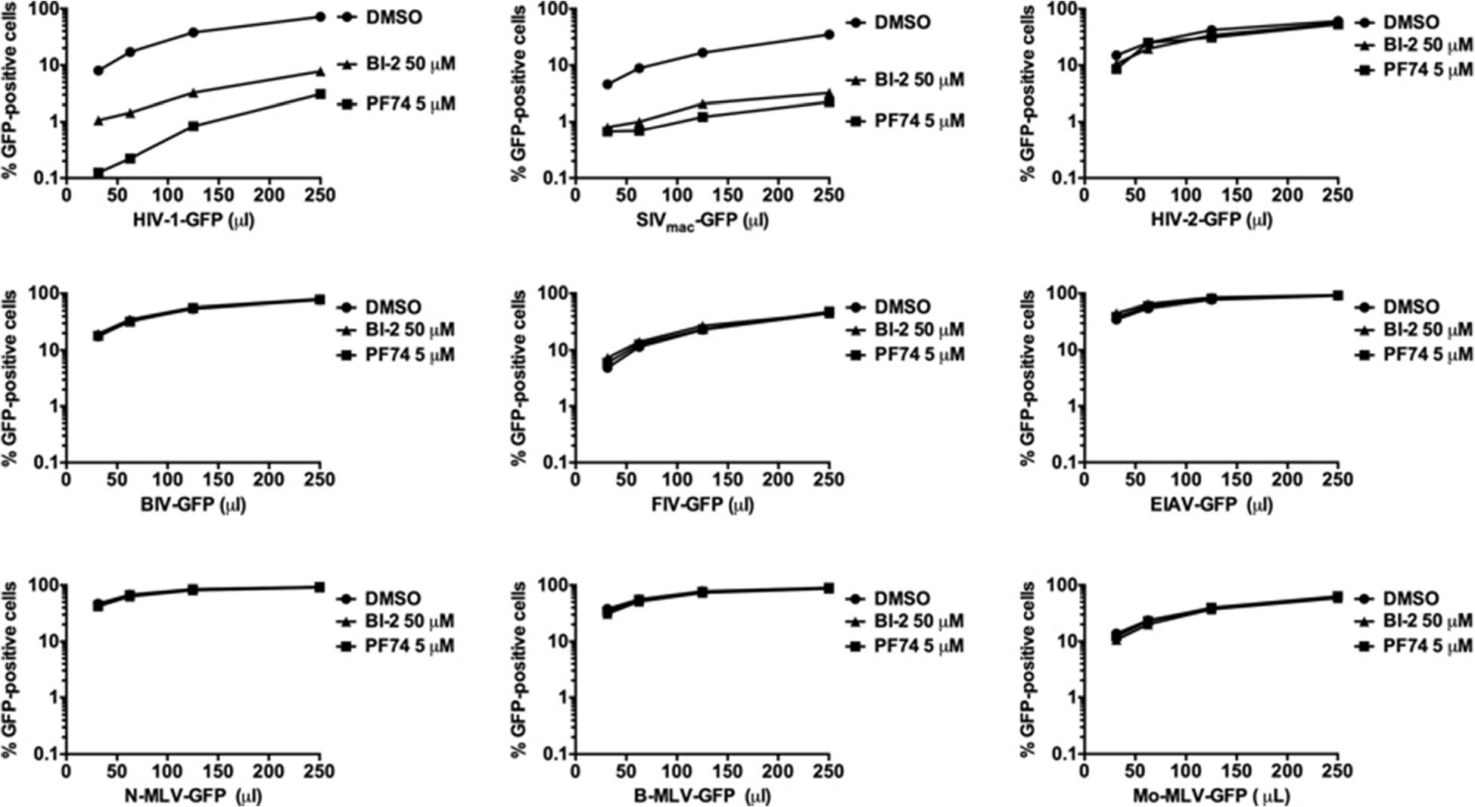
**BI-2 blocks HIV-1 and SIV**_**mac**_ **infection.** Cf2Th cells were challenged with increasing amount of the indicated retrovirus in the presence of BI-2. Infection was determined by measuring the percentage of GFP-positive cells by flow cytometry 48 hours post infection. As control, we performed similar experiments in the presence of the small-molecule PF74. Similar results were obtained in three independent experiments and a representative experiment is shown.

Next we explored the ability of BI-2 to block infection by different retroviruses. For this purpose, we challenged Cf2Th cells with increasing amounts of different retroviruses expressing GFP as reporter of infection (Figure 4), including HIV-1, SIV_mac_, HIV-2_ROD_, bovine immunodeficiency virus (BIV), feline immunodeficiency virus (FIV), equine infectious anemia virus (EIAV), N-tropic murine leukemia virus (N-MLV), B-tropic murine leukemia virus (B-MLV) and Moloney murine leukemia virus (Mo-MLV). Viruses expressing GFP as a reporter were prepared as previously described [23]. Interestingly, BI-2 potently blocked HIV-1 and SIV_mac_ but not HIV-2_ROD_, BIV, FIV, EIAV, N-MLV, B-MLV and Mo-MLV. As a control, we performed similar infections in the presence of PF74 (Figure 4). As previously shown PF74 blocks HIV-1-GFP and SIV_mac_-GFP infection [5,6,17,24]. Interestingly, we found a parallel between the ability of BI-2 to inhibit infection by HIV-GFP and SIV_mac_-GFP with the ability of BI-2 to prevent the binding of CPSF6 with the HIV-1 and SIVmac cores.

This short-form article thoroughly examined and compared the effects of BI-2 and PF74 on HIV-1 infection. Our novel findings demonstrate that BI-2, similar to PF74, destabilizes the HIV-1 core during infection and prevents the binding of CPSF6 to the HIV-1 core.
